# Drug-Delivery Silver Nanoparticles: A New Perspective for Phenindione as an Anticoagulant

**DOI:** 10.3390/biomedicines11082201

**Published:** 2023-08-04

**Authors:** Stoyanka Nikolova, Miglena Milusheva, Vera Gledacheva, Mehran Feizi-Dehnayebi, Lidia Kaynarova, Deyana Georgieva, Vassil Delchev, Iliyana Stefanova, Yulian Tumbarski, Rositsa Mihaylova, Emiliya Cherneva, Snezhana Stoencheva, Mina Todorova

**Affiliations:** 1 Department of Organic Chemistry, Faculty of Chemistry, University of Plovdiv, 4000 Plovdiv, Bulgaria or miglena.milusheva@mu-plovdiv.bg (M.M.); minatodorova@uni-plovdiv.bg (M.T.); 2 Department of Bioorganic Chemistry, Faculty of Pharmacy, Medical University of Plovdiv, 4002 Plovdiv, Bulgaria; 3 Department of Medical Physics and Biophysics, Faculty of Pharmacy, Medical University of Plovdiv, 4002 Plovdiv, Bulgaria; vera.gledacheva@mu-plovdiv.bg (V.G.); iliyana.stefanova@mu-plovdiv.bg (I.S.); 4 Department of Chemistry, Faculty of Science, University of Sistan and Baluchestan, Zahedan P.O. Box 98135-674, Iran; m.feizi@sutech.ac.ir; 5 Department of Analytical Chemistry and Computer Chemistry, Faculty of Chemistry, University of Plovdiv, 4000 Plovdiv, Bulgaria; l.kainarova@uni-plovdiv.bg (L.K.); georgieva@uni-plovdiv.bg (D.G.); 6 Department of Physical Chemistry, Faculty of Chemistry, University of Plovdiv, 4000 Plovdiv, Bulgaria; 7 Department of Microbiology, Technological Faculty, University of Food Technologies, 4002 Plovdiv, Bulgaria; tumbarski@abv.bg; 8 Laboratory of Experimental Chemotherapy, Department “Pharmacology, Pharmacotherapy and Toxicology”, Faculty of Pharmacy, Medical University, 1431 Sofia, Bulgaria; rositsa.a.mihaylova@gmail.com; 9 Department of Chemistry, Faculty of Pharmacy, Medical University of Sofia, 2 Dunav Str., 1000 Sofia, Bulgaria; cherneva@pharmfac.mu-sofia.bg; 10 Organic Chemistry with Centre of Phytochemistry, Bulgarian Academy of Sciences, Acad. G. Bonchev Str., BI 9, 1113 Sofia, Bulgaria; 11 University Hospital “Sveti Georgi” EAD, 4002 Plovdiv, Bulgaria; 12 Department of Clinical Laboratory, Faculty of Pharmacy, Medical University of Plovdiv, 4002 Plovdiv, Bulgaria

**Keywords:** silver nanoparticles, galactose-assisted, phenindione (2-phenyl-1,3-indandione), density functional theory (DFT), anticoagulant, thrombosis, antimicrobial, cytotoxicity, spasmolytic

## Abstract

Anticoagulants prevent the blood from developing the coagulation process, which is the primary cause of death in thromboembolic illnesses. Phenindione (PID) is a well-known anticoagulant that is rarely employed because it totally prevents coagulation, which can be a life-threatening complication. The goal of the current study is to synthesize drug-loaded Ag NPs to slow down the coagulation process. Methods: A rapid synthesis and stabilization of silver nanoparticles as drug-delivery systems for phenindione (PID) were applied for the first time. Results: Several methods are used to determine the size of the resulting Ag NPs. Additionally, the drug-release capabilities of Ag NPs were established. Density functional theory (DFT) calculations were performed for the first time to indicate the nature of the interaction between PID and nanostructures. DFT findings supported that galactose-loaded nanostructure could be a proper delivery system for phenindione. The drug-loaded Ag NPs were characterized in vitro for their antimicrobial, cytotoxic, and anticoagulant activities, and ex vivo for spasmolytic activity. The obtained data confirmed the drug-release experiments. Drug-loaded Ag NPs showed that prothrombin time (PT, sec) and activated partial thromboplastin time (APTT, sec) are approximately 1.5 times longer than the normal values, while PID itself stopped coagulation at all. This can make the PID-loaded Ag NPs better therapeutic anticoagulants. PID was compared to PID-loaded Ag NPs in antimicrobial, spasmolytic activity, and cytotoxicity. All the experiments confirmed the drug-release results.

## 1. Introduction

The vascular system must be in a homeostatic state for coagulation and fibrinolytic processes to function properly. These processes are crucial to the protective physiological systems of the organism. In healthy individuals, microscopic blood clots are frequently required for the repair of vascular injuries. The fibrinolytic system effectively eliminates insoluble clots by breaking them down into soluble fragments through proteolytic digestion and repairing the damaged vessel’s endothelial surface [1]. Anticoagulants prevent the blood from developing the coagulation process. Anticoagulant therapy is focused on preventing blood vessel clot formation, which is the primary cause of death in thromboembolic illnesses [2,3,4]. Orally ingestible medications are used in standard therapeutic protocols for the prophylaxis and treatment of venous thrombosis and to lower the risk of recurrent myocardial infarction [5,6]. In order to achieve more effective therapeutic results, combination therapy with anticoagulants and platelet aggregation inhibitors is routinely utilized in high-risk patients [7,8]. However, these medications have some undesirable side effects, such as aspirin-related gastrointestinal ulcers, allergy-related bleeding, or warfarin-induced skin necrosis [6,9]. Furthermore, the effectiveness of these medications is still not adequate. Therefore, medicinal chemists continue trying to find new drug candidates in this therapeutic area [10]. Among the known oral anticoagulants, coumarin, warfarin, and indanone derivatives are well distributed. Their therapeutic action depends on their ability to suppress the formation of several functional factors of blood coagulation in the liver [1,11].

Numerous medications have an impact on platelet and coagulation activity. They might work by amplifying the innate inhibition of coagulation, especially at the level of thrombin and factor Xa. Many oral anticoagulants work by inhibiting vitamin K, which prevents the formation of factors II, VII, and X, protein C, and other proteins that are necessary for blood clotting. The more popular oral anticoagulants are vitamin K-like coumarin derivatives, such as warfarin, dicoumarides, and phenindione (2-phenyl-1,3-indandione, PID). Clinically, warfarin is used to reduce the incidence of thromboembolism associated with prosthetic heart valves and atrial fibrillation. PID’s effects are comparable to those of warfarin, but it is no longer often used due to the risk of serious side effects [1,11,12,13,14]. 

Indane and its analogues present a variety of biological applications by serving as pharmacophores and by providing scaffolds for the rational design of medications deliberated at diverse biological targets [15]. The indanes’ bioactive profile stimulated the creation of therapeutically effective medications and therapeutically useful molecules. Indanes enhance the molecular interactions with target peptides and enzymes, enabling the efficient modulation of enzyme expression to yield the optimal pharmacological effect [16]. Indanes are effective scaffolds for the synthesis of anticancer and anti-inflammatory compounds [17,18,19]. The bioactive compounds based on these scaffolds allow for the targeting of composite metastatic pathways, providing an additional benefit for treating malignancies with multiple subtypes. Similarly, indanes and indanones form the core structure of several bioactive natural compounds and secondary metabolites with radical scavenging properties that serve as a blueprint for the structural optimization of bioactive indane-based synthetic compounds [20]. 

The indanone nucleus, its analogs, and derivatives offer intriguing biological uses and medicinally significant molecules that are therapeutically effective. Indanone-based medications provide an expeditious profile in the current drug development paradigm focused on anti-inflammatory and anti-Alzheimer therapies. Indane nucleus is a successful medication for Parkinson’s disease treatment. The indanone structural motif is applied in the treatment of pulmonary embolism, mural thrombosis, and cardiomyopathy or serves as an anticoagulant [21]. Therefore, indanes are strong candidates for molecules in the modern drug development paradigm.

Silver nanoparticles (Ag NPs), on the other hand, possess anticoagulant and anti-inflammatory activity, which makes them ideal candidates for biological applications [22,23,24,25,26,27,28,29,30]. Finding an environmentally friendly method to make NPs is the main challenge in contemporary synthetic organic chemistry [31,32,33]. The noble metal cations can be converted to their nano-forms using biological procedures that utilize various biological resources as reducing and stabilizing agents [34,35,36,37]. Polymer-based NPs were synthesized due to the spectrum of biological interest [38]. Recently, the application of carbohydrates has become a popular area in NP synthesis [39,40,41]. Sugars as reducing agents for the green synthesis of metal NPs have been applied in the last ten years [42,43,44,45,46]. Many mono-, di-, and polysaccharides were used as reducing and capping agents for the preparation of noble metal NPs due to their sustainability, abundance, low cost, harmlessness, renewability, biodegradability, and compatibility with biological systems [47,48,49,50,51]. The capping agents have an effective role in NP’s growth and control of their size but could be hazardous [52], causing many doubts about their use in biological applications [53]. 

Bearing in mind the structural moiety of indanones and Ag NPs and their biological activities, novel Ag NPs as drug-delivery systems for PID were obtained. The synthesized drug-loaded Ag NPs could be effective as a new therapeutic anticoagulant in both thrombosis treatment and medical device coating.

## 2. Materials and Methods

All solvents and reagents were purchased from Merck (Merck Bulgaria EAD, Sofia, Bulgaria). The melting point was determined on a Boetius hot stage apparatus and is uncorrected. Elemental analyses were performed with a TruspecMicro (LECO, Mönchengladbach, Germany). PID was characterized using IR, ^1^H-NMR, and ^13^C-NMR. IR spectra were determined on a VERTEX 70 FT-IR spectrometer (Bruker Optics, Ettlingen, Germany). ^1^H-NMR and ^13^C-NMR spectra were recorded on a Bruker Avance III HD 500 spectrometer (Bruker, Billerica, MA, USA) at 500 MHz (^1^H-NMR) and 125 MHz (^13^C-NMR), respectively. Chemical shifts are given in relative ppm and were referenced to tetramethylsilane (TMS) (δ = 0.00 ppm) as an internal standard; the coupling constants are indicated in Hz. The NMR spectra were recorded at room temperature (ac. 295 K). The purity was determined by TLC using several solvent systems of different polarities. TLC was carried out on precoated 0.2 mm Fluka silica gel 60 plates (Merck Bulgaria EAD), using chloroform:n-hexane:methanol: acetone = 4:4:1:1 as a chromatographic system. 

### 2.1. Synthetic Methods

#### 2.1.1. Synthesis of 2-Phenyl-1,3-Indandione

**Scheme 1:**
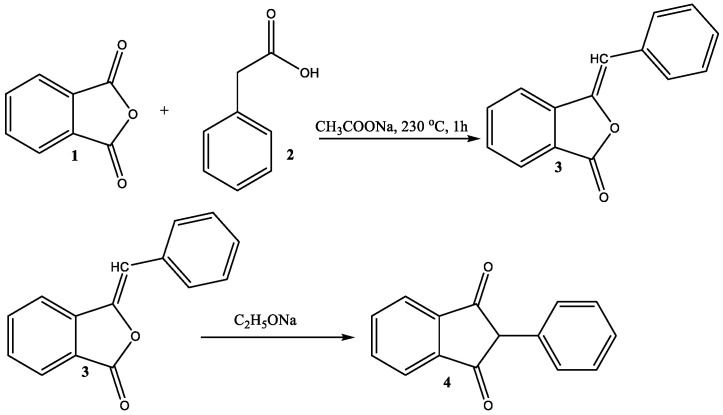
Synthesis of phenindione (PID).

2-phenyl-1,3-indandione is synthesized using a known procedure, via condensation of phenylacetic acid **2** with phthalic anhydride **1** to phenylmethylenphthalide **3**, and further rearrangement in the presence of sodium ethoxide to PID **4** [54] (Scheme 1). 

2-phenyl-1H-indene-1,3(2H)-dione (**4**): ^1^H-NMR: 4.29 (s, 1H, CH), 7.21–7.22 (m, 2H, Ar), 7.31–7.39 (m, 3H, Ar), 7.91–7.98 (dd, J = 10, 5, 2H, Ar), 8.06–8.10 (dd, J = 10, 5, 2H, Ar); ^13^C-NMR: 198.3 (CO), 142.7, 136.0, 133.2, 129.0, 128.8, 123.8, 59.8 (CH). IR [KBr]: ν, cm^−1^ 3088, 3078, 3025, 3006 (ν C-H (Ar-H)), 2888 (ν (CH), methine group), 1746, 1705 (ν (C=O)), 1582, 1498, 1473, 1453 ν( CC ) Ar, 1344 (δ(CH), methine group), 768 (γ C-H(Ar-H)); Anal. calcd. for C_15_H_10_O_2_: C 81.07; H 4.54; Found: C 81.10; H 4.57 (Supplementary Materials Figures S1–S3).

#### 2.1.2. Synthesis of Galactose-Assisted PID-Loaded Ag NPs

A 0.01 M AgNO_3_ solution was added to 1.25 g (0.007 mol) of galactose that had been dissolved in 25 mL of water and refluxed for 2 min. Different concentrations of PID were added to the solutions to examine how the ratio of Ag NPs to the drug molecule would impact drug release and biological activity. Galactose and AgNO_3_ concentrations were constant during the course of the trials, whereas medication concentrations varied based on the ratio. Drug molecules were arranged in the following ratios to Ag NPs: 1:1, 1:5, 1:10, 1:20, and 1:50 (concentration range from 4 × 10^−4^ to 2 × 10^−2^ mol/L). In about 5 min, the color of the solution turned pale yellow, indicating the formation of Ag NPs. 

### 2.2. Characterization of the Ag NPs. Analytical Techniques

The solution was utilized for UV-Vis, TEM, single particle ICP-MS (sp-ICP-MS), dynamic light scattering (DLS), and zeta potential. The suspension was centrifuged at 5000 rpm for 15 min, filtered (0.22 m, Chromafil^®^, Macherey-Nagel, Düren, Germany), and the precipitate was used for Fourier transform infrared spectra (FTIR) and X-ray diffraction (XRD) analyses.

#### 2.2.1. UV-Vis Spectra

UV–Vis spectra were recorded in the range between 320 and 800 nm using a Cary-60 UV-Vis spectrophotometer (Agilent Technologies, Santa Clara, CA, USA). The produced nanoparticle dispersions’ absorbance was measured in a quartz cuvette with a 1 cm optical path.

#### 2.2.2. FTIR Spectra

IR spectra were determined on an FT-IR spectrometer VERTEX 70. The spectra were collected in the range from 600 cm^−1^ to 4000 cm^−1^ with a resolution of 4 nm and 20 scans. The instrument is equipped with a diamond-attenuated total reflection (ATR) accessory. The IR spectra were analyzed with the OPUS-Spectroscopy Software, Bruker (Version 7.0, Bruker, Ettlingen, Germany).

#### 2.2.3. spICP-MS

A 7700 Agilent ICP-MS spectrometer equipped with a MicroMist™ nebulizer and Peltier-cooled double-pass spray chamber was used for the characterization of silver nanoparticles at 107 amu. The ICP-MS operating parameters are as follows: RF power—1.55 kW; sample flow rate—0.322 mL min^−1^; carrier Ar gas flow rate—1.2 L min^−1^; acquisition time 60 s; dwell time 5 ms; and transport efficiency 0.032. The transport efficiency was determined by using the particle-size method [55]. Ultrapure water (UPW) was used throughout the experiments (PURELAB Chorus 2+ (ELGA Veolia, High Wycombe, UK) water purification system). For the sonication of silver colloids, an ultrasonic bath (Kerry US, Burlingame, CA, USA) was used. Reference materials (RM) of citrate-stabilized silver dispersions Ag NPs (Sigma-Aldrich, Saint Louis, MO, USA) with mean size 40 ± 4 nm and total mass concentration of silver 0.02 mg mL^−1^ were used in this study for transport efficiency determination and calibration.

A standard solution of Ag 9.974 ± 0.041 mg L^−1^ in 2% HNO_3_, (CPAchem Ltd., Bogomilovo, Bulgaria) was used for the preparation of ionic standards. 

#### 2.2.4. TEM

The morphology and size of drug-loaded Ag NPs were also observed using TEM. A drop of the nanosuspension was placed on a 200 mesh formvar-coated copper grid and allowed to dry for 24 h. Images were obtained using Talos F200C G2 Transmission Electron Microscope (Talos 1.15.3, Thermo Fisher Scientific, Waltham, MA, USA) operating at 200 kV and analyzed using Velox Imaging Software (Velox 2.15.0.45, Waltham, MA, USA).

#### 2.2.5. DLS and Zeta Potential

DLS measurements were carried out on a Brookhaven BI-200 goniometer with vertically polarized incident light at a wavelength of l = 632.8 nm supplied by a He–Ne laser operating at 35 mW and equipped with a Brookhaven BI-9000 AT digital autocorrelator. The scattered light was measured for dilute aqueous dispersions in the concentration range 0.056–0.963 mg mL^−1^ at 25, 37, and 65 °C. Measurements were made at angles ζ in the range of 50–130°. The system allows measurements of ζ-potential in the range from −200 mV to +200 mV. All analyses were performed in triplicate at 25 °C.

#### 2.2.6. X-ray Diffraction (XRD)

The level of crystallinity of the synthesized nanoparticles was studied by using X-ray powder diffractometry. Using a SIEMENS D500 X-ray powder diffractometer (KS Analytical Systems, Aubrey, TX, USA), the diffraction patterns of Ag NPs (blank) and drug-loaded Ag NPs were recorded at a 2θ range from 10° to 80°. All measurements were carried out at a 35 kV voltage and a 25 mA current. Using a Cu-anticathode (K1), monochromatic X-rays (1.5406) were produced. 

### 2.3. In Vitro Drug Release

Using the dialysis bag approach, in vitro drug release was evaluated. A dialysis membrane was hydrated in distilled water for 24 h (MWCO 12 kDa, Sigma-Aldrich, St. Louis, MO, USA). Drug-loaded Ag NPs (equivalent to the amount of PID in ratios of 1:1, 1:5, 1:10, 1:20, and 1:50) were dispersed in 10 mL of phosphate-buffered saline (PBS) and then transferred to the dialysis bag, closed with a plastic clamp. Each bag was placed into a beaker with 40 mL of PBS (dialysis medium, pH 7.4). Aliquots with 2 mL of each dialysis medium were taken for measurements, and then the fresh medium was added at specified intervals. The drug release experiment lasted 24 h. The mean results of triplicate measurements and standard deviations were reported. The solution in the Falcon tube was shaken before each evaluation of UV–visible absorbance. For data analysis, PID’s maximum absorption band values (λ = 274, 230, and 204 nm) were employed. Additionally, drug-only controls (one drug control for each ratio) were made.

### 2.4. Computational Perspective

In the current study, the 3D structure of PID and the surface of nanoparticle containing three galactose molecules were built and optimized by Gauss View 6.0 and Gaussian 09W package [56] for delivering the PID as an anticoagulant. The density functional theory (DFT) approach was carried out utilizing the B3LYP method with the 6–311 g++(d,p) basic set for all atoms. All these calculations were conducted at the ground state in the gas phase. The harmonic frequencies of all the structures were checked for positivity to determine that they were at their true minimum. The aim was to obtain optimized geometries, molecular electrostatic potential (MEP) surfaces, HOMO (highest occupied molecular orbital)-LUMO (lowest unoccupied molecular orbital) analysis and determine the type of interaction between PID and nanoparticle-containing three galactoses. Using the following formula, the *E_ads_* (adsorption energy) of PID on the surface of nanoparticle containing galactose was estimated [57]:

$$E_{ads}=\left(E_{PID\;on\;nanostructure}\right)-\left(E_{nanostructure}+E_{PID}\right)$$

Herein, *E_PID on nanostructure_*, *E_nanostructure_*, and *E_PID_* are denoted as the total energy of PID on the nanostructure, the primal energy of free nanostructure, and the primal energy of free PID, respectively.

### 2.5. Microbiological Tests

**Tested Microorganisms**

Twenty tested microorganisms, including six Gram-positive bacteria (*Bacillus subtilis* ATCC 6633, *Bacillus amyloliquefaciens* 4BCL-YT, *Staphylococcus aureus* ATCC 25923, *Listeria monocytogenes* NBIMCC 8632, *Enterococcus faecalis* ATCC 19433, and *Micrococcus luteus* 2YC-YT), six Gram-negative bacteria (*Salmonella enteritidis* ATCC 13076, *Klebsiella* sp.—clinical isolate, *Escherichia coli* ATCC 25922, *Proteus vulgaris* ATCC 6380, *Pseudomonas aeruginosa* ATCC 9027, *Salmonella typhimurium* NBIMCC 1672), two yeasts (*Candida albicans* NBIMCC 74, *Saccharomyces cerevisiae* ATCC 9763) and six fungi (*Aspergillus niger* ATCC 1015, *Aspergillus flavus*, *Penicillium* sp., *Rhizopus* sp., *Mucor* sp.—plant isolates, *Fusarium moniliforme* ATCC 38932) from the collection of the Department of Microbiology at the University of Food Technologies—Plovdiv, Bulgaria, were selected for the antimicrobial activity test. 

Culture media

Luria-Bertani agar medium supplemented with glucose (LBG agar) 

LBG agar was prepared according to the manufacturer’s (Laboratorios Conda S.A., Madrid, Spain) prescription: 50 g of LBG-solid substance mixture (containing 10 g tryptone, 5 g yeast extract, 10 g NaCl, 10 g glucose, and 15 g agar) was dissolved in 1 L of deionized water (pH 7.5), and the then medium was autoclaved at 121 °C for 20 min.

Malt extract agar (MEA)

MEA was prepared by the manufacturer’s (HiMedia^®^, Mumbai, India) prescription: 50 g of the MEA-solid substance mixture (containing 30 g malt extract, 5 g mycological peptone, and 15 g agar) were dissolved in 1 L of deionized water (pH 5.4), and then the medium was autoclaved at 115 °C for 15 min.

Antimicrobial activity assay

The antimicrobial activity of the samples was determined by using the agar-well diffusion method. The tested bacteria *B. subtilis* and *B. amyloliquefaciens* were cultured on LBG agar at 30 °C. The tested bacteria *S. aureus*, *L. monocytogenes*, *E. faecalis*, *S. enteritidis*, *Klebsiella* sp., *E. coli*, *P. vulgaris,* and *P. aeruginosa* were cultured on LBG agar at 37 °C for 24 h. The yeast *C. albicans* was cultured on MEA at 37 °C, while *S. cerevisiae* was cultured on MEA at 30 °C for 24 h. The fungi *A. niger*, *A. flavus*, *Penicillium* sp., *Rhizopus* sp., *Mucor* sp., and *F. moniliforme* were grown on MEA at 30 °C for 7 days or until sporulation. 

The inocula of the tested bacteria/yeasts were prepared by homogenization of a small amount of biomass in 5 mL of sterile 0.5% NaCl. The inocula of tested fungi were prepared by the addition of 5 mL of sterile 0.5% NaCl into the tubes. After stirring by vortex V-1 plus (Biosan, Riga, Latvia), they were filtered and replaced in other tubes before use. The number of viable cells and fungal spores was determined using a bacterial counting chamber Thoma (Poly-Optik, Görlitz, Germany). Their final concentrations were adjusted to 10^8^ cfu/mL for bacterial/yeast cells and 10^5^ cfu/mL for fungal spores and then inoculated in preliminarily melted and tempered at 45–48 °C LBG/MEA agar media. Next, the inoculated media were transferred in a quantity of 18 mL in sterile Petri plates (d = 90 mm) (Gosselin™) and allowed to harden. Then six wells (d = 6 mm) per plate were cut, and triplicates of 60 μL of each extract were pipetted into the agar wells. The Petri plates were incubated at identical conditions.

The antimicrobial activity was determined by measuring the diameter of the inhibition zones around the wells on the 24th and 48th hour of incubation. Tested microorganisms with inhibition zones of 18 mm or more were considered sensitive; moderately sensitive were those in which the zones were from 12 to 18 mm; resistant were those in which the inhibition zones were up to 12 mm or completely missing [58]. 

### 2.6. Cytotoxic Activity

In order to evaluate the in vitro biocompatibility of the PID-loaded Ag NPs, a series of cell viability assays were performed against human malignant cell lines of hematological (bcr-abl positive LAMA-84 and K-562 chronic myeloid leukemia cells) and epithelial (T-24 urothelial bladder carcinoma cells) origin, as well as normal murine fibroblast cells (CCL-1). All cell lines were purchased from the German Collection of Microorganisms and Cell Cultures (DSMZ GmbH, Braunschweig, Germany). Cell cultures were cultivated in a growth medium RPMI 1640 supplemented with 10% fetal bovine serum (FBS) and 5% L-glutamine and incubated under standard conditions of 37 °C and 5% humidified CO_2_ atmosphere.

Cell viability assay

The experimental design involved several cytotoxicity assays that measured cell growth inhibition by the newly synthesized drug-loaded Ag NPs. Cell viability was evaluated using a standard MTT-based colorimetric assay. Exponential-phased cells were harvested and seeded (100 μL/well) in 96-well plates at the appropriate density (3 × 10^5^) for the suspension cultures (LAMA-84 and K-562) and 1.5 × 10^5^ for the adherent ones (CCL-1, T-24). Cells were treated and incubated with various concentrations of the experimental compounds in the concentration range of 400–6.25 µM. After an exposure time of 72 h, filter-sterilized MTT substrate solution (5 mg/mL in PBS) was added to each well of the culture plate. A further 1–4 h incubation allowed for the formation of purple, insoluble formazan precipitates. The latter was dissolved in isopropyl alcohol solution containing 5% formic acid prior to absorbance measurement at 550 nm using a microplate reader (Labexim LMR-1). Collected absorbance values were blanked against MTT and isopropanol solution and normalized to the mean value of untreated control (100% cell viability). Semi-logarithmic “dose-response” curves were constructed, and the half-inhibitory concentrations of the screened compounds against each tested cell line were calculated. Values of *p* ≤ 0.05 were considered statistically significant.

### 2.7. Anticoagulant Activity

Platelet-poor plasma is used for screening coagulation tests and markers of activation of coagulation. It is obtained by centrifugation of the blood with sodium citrate at 3000–3500 U/min and 2000× *g* for 10–15 min. Prothrombin time (PT) activated partial thromboplastin time (APTT), and fibrinogen was immediately analyzed on an automated coagulation analyzer Sysmex SC 2000i (Siemens Corporation, Wakinohama-Kaigandori, Chuo-Ku, Kobe 651-0073, Japan). Fibrinogen was measured by von Clauss chronometric method [59].

### 2.8. Smooth Muscle Activity

#### 2.8.1. Ex Vivo Experiments on Gastric Smooth Muscle Preparations (SMPs) from Rat Wistar

SMPs with dimensions 1.0–1.5 mm wide and 10–12 mm long were obtained from adult male Wistar rats weighing about 270 g. Strips were circularly dissected from corpus gastric muscle and mounted in a tissue bath and superfused with warmed (37 °C) Krebs solution. The number of SM preparations used for each data point is indicated by n.

The pH of the solution was measured before each experiment by a pH meter, HI5521 (Hanna Instruments, Woonsocket, RI, USA). Krebs bathing solution was continuously aerated with a mixture of 95% O_2_ and 5% CO_2_. Krebs contained the following (in mmol/L): NaCl 120; KCl 5.9; CaCl_2_ 2.5; MgCl_2_ 1.2; NaH_2_PO_4_ 1.2; NaHCO_3_ 15.4; glucose 11.5 at pH 7.4. 

#### 2.8.2. Method of Studying a Mechanical Activity of Isolated SM Preparation

The contractile activity (CA) of the SMPs and the changes in substance-evoked reactions were detected isometrically by using Tissue Organ Bath System 159,920 Radnoti (Dublin, Ireland). The initial mechanical stress of the preparations obtained by the stretch tension system corresponded to a tensile force of 10 mN. SM tissue vitality was tested by adding 1 × 10^−6^ mol/L Ach at baseline. The tissue was equilibrated for 60 min and washed every 15–20 min by replacing the Krebs solution. In the meanwhile, the compounds were prepared for the experiment. This requires a more concentrated solution than the actual concentration in the bath, so only a small volume (1/100) of the drug stock was needed to achieve the desired concentration. Then, an agonist was picked (a compound that causes active contraction) to which the tissue responds. The intactness of the contractile apparatus of SMPs during and at the end of experiments was checked by adding 1 × 10^−6^ mol/L Ach between each treatment with drugs. 

### 2.9. Ethics Statement

Animals used in experiments were male Wistar rats. The experiments were approved by the Ethical Committee of the Bulgarian Food Agency with No 229/09.04.2019 and were carried out following the guidelines of the European Directive 2010/63/EU. The animals were provided by the Animal House of Medical University Plovdiv, Bulgaria.

### 2.10. Statistical Analysis

The Instat computer program for analysis of the variance was used. The mean and standard error of the mean (SEM) for each group were calculated. A two-way ANOVA for repeated measurements was used to compare different groups with the respective controls. A *p*-value of *p* < 0.05 was considered representative of a significant difference.

IBM SPSS Statistics v. 26 statistical package was used for statistical analyses.

## 3. Results and Discussion

Drug-loaded Ag NPs were synthesized using a previously described procedure under green one-pot reaction conditions: in water as a solvent, with no organic solvents, and at the boiling point of water [44]. Anhydrous AgNO_3_ was used as a precursor for Ag NPs. The capping and reducing agent that can quickly reduce Ag^+^ to the ground state at the boiling point of water (100 °C) was galactose [44]. Galactose-capped Ag NPs were chosen due to their lower toxicity [60,61], biocompatibility, and suitability for exploitation in medical applications. PID was used as a compound with known anticoagulant activity. 

### 3.1. Physical Characterizations of Galactose-Assisted Drug-Loaded Ag NPs

**Figure 1:**
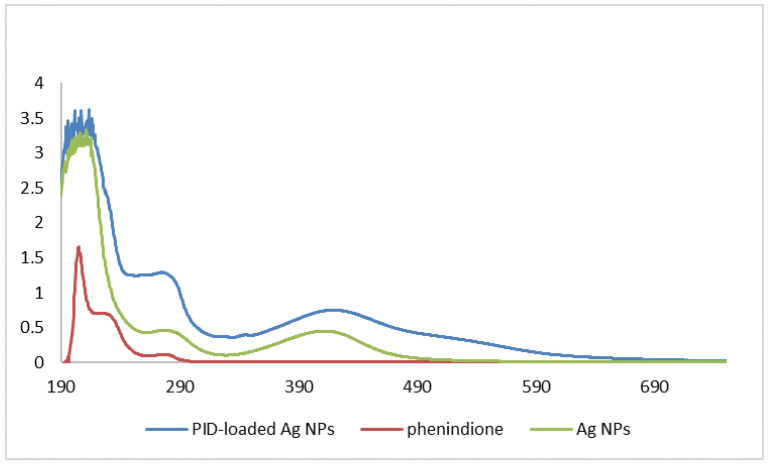
Absorption spectra of PID (red), AgNPs (light green), and PID-loaded Ag NPs (blue).

Initially, the synthesis of drug-loaded Ag NPs was monitored by recording the UV–visible spectra in the region 190–800 nm (Figure 1). The absorption maximum in galactose solution was detected at 287 nm, while PID was observed at 274, 230, and 204 nm. The appearance of a surface plasmon resonance (SPR) at 418 nm showed the formation of the Ag NPs [41,62]. The SPR property of metallic NPs is one of the most important characteristics, which depends on the size and shape of synthesized metals [63].

The position of the SPR in the visible part of the spectrum also serves as a reference for the shape of the nanoparticles [64,65,66,67,68]. The wavelength of the SPR peak is in the region 410–450 nm, which is indicative of the spherical shape of the obtained nanoparticles [41,69,70].

UV–visible spectroscopy is used also to investigate Ag NP colloidal aggregation [71]. In our previous study, we observed a symmetric plasmon band for the drug-loaded Ag NPs, which corresponds to their low degree of aggregation [41]. In the present study, the observed plasmon band is rather asymmetric. A possible explanation is the existence of two tautomeric forms for PID.

**Figure 2:**
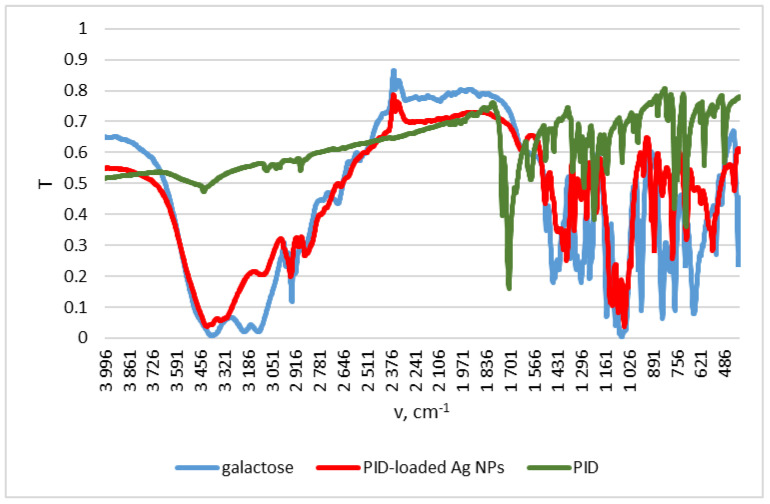
IR spectra of PID, PID-loaded Ag NPs, and galactose in the region of 4000–400 cm^−1^.

The synthesis of drug-loaded Ag NPs was also investigated by using FTIR and compared to any of the reactants (Figure 2). IR analysis depicted shifts in the characteristic peaks of galactose, Ag NPs, and PID-loaded Ag NPs indicating interactions amongst the molecules. 

IR analysis depicted the changes resulting from modified galactose-assisted PID-loaded Ag NPs carrier functionality due to incorporating silver nanoparticles. The participation of the carbohydrate in the synthesis of the nanoparticles is confirmed by the observed band shifts in the IR spectrum of drug-loaded Ag NPs compared to pure galactose [72]. Certain functional groups, such as C-O and glycosidic hydroxyl groups, have a significant contribution to the fixation of nanoparticles [73].

Wiercigroch et al. separated the IR spectra of galactose into five regions [74]. According to the authors, the first region involves deformation vibration (β) of the pyranose ring, In the pyranose ring of galactose, stretching vibrations v(C-O), (C-C), and in-plane bending (COH) were seen at 1151, 1104, and 1045 cm^−1^ [75], respectively, whereas shifts were seen for the PID-loaded Ag NPs at 1211, 1121, 1119, 1094, 1069, and 1051 cm^−1^. The presence of an α-anomer is shown by an 837 cm^−1^ band in both galactose and PID-loaded Ag NPs IR spectra [76,77]. The bands corresponding to the vibrations of the alpha and beta anomeric forms of galactose as well as the vibrations of the pyranose ring are observed in the second region of the infrared spectrum. The bands that correspond to the hydroxyl group vibrations are in the fifth region. The bands for the C-H stretching vibrations of the methylene and methine groups are observed in the fourth region, while the bands for the deformation C-H vibrations of the methylene groups are located in the third area. A stretching vibration for the hydroxyl groups in the galactose spectrum appeared at 3386 cm^−1^, 3205 cm^−1^, and 3131 cm^−1^, while shifts were observed at 3390 cm^−1^ and 3216 cm^−1^ for PID-loaded Ag NPs. The bands for the stretching C-H vibration ν(C-H) in the galactose molecule appeared at 2949 cm^−1^, 2937 cm^−1^, and 2916 cm^−1^. Four bands were observed for drug-loaded Ag NPs at 2939, 2867, 2863, and 2849 cm^−1^. The bands for the deformation vibrations of the CH_2_ group in the IR spectrum of galactose are observed in the area 1457–1248 cm^−1^, and a shift from 1496 cm^−1^ to 1238 cm^−1^ was observed for PID-loaded Ag NPs. 

Ag NPs were synthesized in the presence of [Ag(NH_3_)_2_]^+^ with the assistance of carbohydrates [41,78]. It is well known that biomolecules interact with the silver’s upper face, where the initial bond to the metal was formed [79,80]. The size and rate of aggregation are decreased by silver nanoparticle coating [81]. The dynamic surface location of the silver nanoparticles allowed the measurement of particle size, shape, and accumulation rate. The reaction parameters, which include temperature, pH, reactant concentration, and duration, have an impact on the shape and size of the silver accumulating [82]. Silver nanoparticles with less than 10 nm diameter have the potential to enter the nuclear cavity and interact with genetic material. Cytotoxicity of nanoparticles relies on their structure; for example, plate-like shape Ag NPs are more dangerous than those with a wire or spherical shape [83,84,85,86].

To establish the size and shape of the Ag NPs, spICP-MS, DLS, and Zeta potential were used.

The nanocolloid suspensions were examined by using spICP-MS to determine the size and distribution by size of the silver core in synthesized PID-loaded Ag NPs. The investigated parameters of the particles, such as mean, mode, and median diameter, as well as size distribution histogram, were evaluated by using an ionic calibration strategy [55]. For this purpose, a set of Ag^+^ ionic standard solutions in 0.5% HNO_3_ with a concentration range of 45–1200 ng L^−1^ were prepared. The levels of calibration standards were selected so that the mass of the delivered silver for chosen dwell time corresponded to Ag NPs with diameters in the range of 20–50 nm. 

Reference material of citrate-stabilized Ag NPs with certified size 40 ± 4 nm and one ionic standard solution of Ag^+^ (1 ng L^−1^) were used for determination of the transport efficiency and instrumental sensitivity. The transport efficiency was calculated by the particle-size method [55].

**Figure 3:**
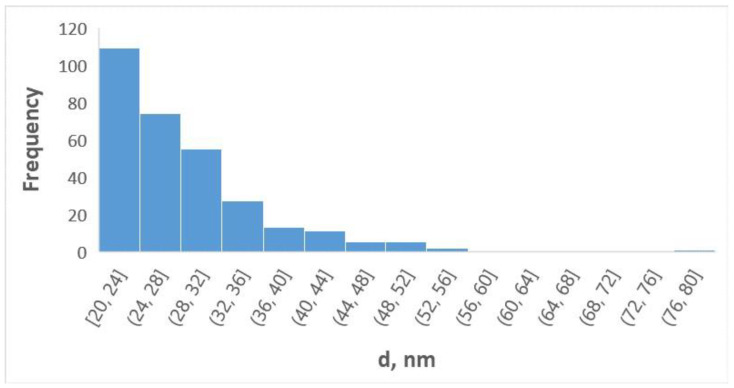
Particle-size distribution histogram of galactose-assisted Ag NPs (~300 NPs).

All measurements were made at the following parameters: dwell time 5 ms, acquisition time 60 s, sample flow rate 0.322 mL·min^−1^, and transport efficiency 0.061. The size histogram of the analyzed NPs is presented in Figure 3.

Based on the obtained histogram, it can be concluded that Ag NPs have an asymmetrical size distribution, with the highest NPs fraction between 20 and 24 nm, which is close to the method’s detection limit—LOD_size_ 17 nm. The estimated mean diameter, mode, and median are as follows: 28 nm, 22 nm, and 27 nm. 

**Figure 4:**
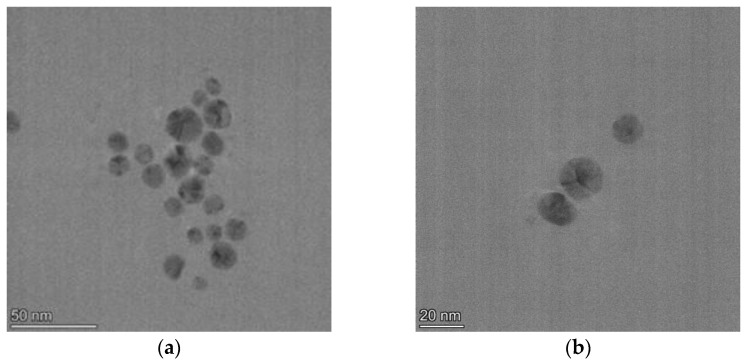
TEM images of galactose-assisted PID-loaded Ag NPs (**a**,**b**).

The results from the size distribution histograms (Figure 3) were confirmed by the TEM images (Figure 4a,b). The obtained data clearly show the size and form of the nanoparticles as well as the individual nanoparticles. The TEM image confirmed the synthesis of spherical particles of different sizes for drug-loaded Ag NPs (Figure 4). We assume that galactose, as well as the benzene ring in PID-loaded Ag NPs (Figure 4a,b), prevent the aggregation of the particles.

**Figure 5:**
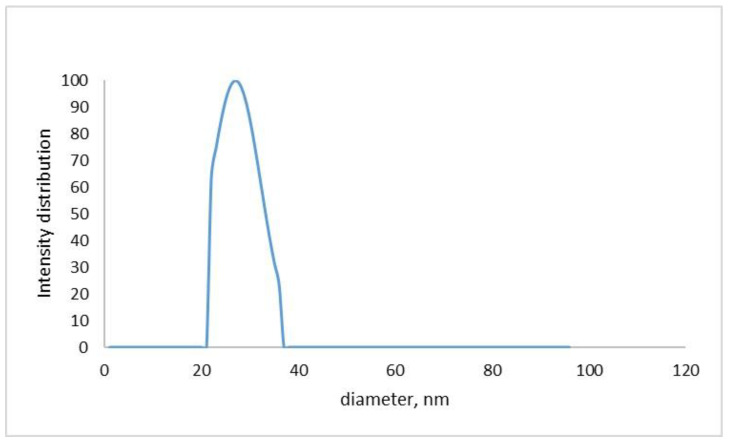
Dynamic light scattering histograms of PID-loaded Ag NPs.

Based on the DLS, the median average size of the obtained particles was identified in the range of 20 to 40 nm for PID-loaded Ag NPs (Figure 5).

For the complex production of silver, many groups, including hydroxyl, carboxyl, phenol, and carbonyl, are associated with oxygen and nitrogen by covalent bond linkage; as a result, they are likely absorbed on their surface [80]. Using NMR and DFT calculations, Sigalov [87] examined the PID for keto-enol tautomerism. According to the estimated data, a strong hydrogen bond contributes to the enol form’s stability in the DMSO solution. It was suggested that the strong Ionic interaction with an anion in the enol forms promotes a rapid proton transfer between carbonyl oxygen atoms, which is the reason for the symmetry of their NMR spectra. It was shown that during keto-enol tautomerization, the 2-phenyl-1,3-indandione in its di-keto form interacts with an anion similarly. That was the reason to assume that the PID’s enol-form can form hydrogen bonds to galactose OH groups while connecting to galactose coating.

The zeta potential of drug-loaded Ag NPs was −12.28 ± 0.28 mV. These results are close to previously described Ag NPs [41]. Carboxylic acids, as the oxidized products of sugars, are utilized to provide a negative surface charge density to counteract the Van der Waals forces responsible for particle coalescence. The carboxylic groups of generated galactonic acid could be a source of the negative charge of the zeta potential. To stabilize metal surfaces, self-assembling carboxylic acids ensure a dense covering [88].

**Figure 6:**
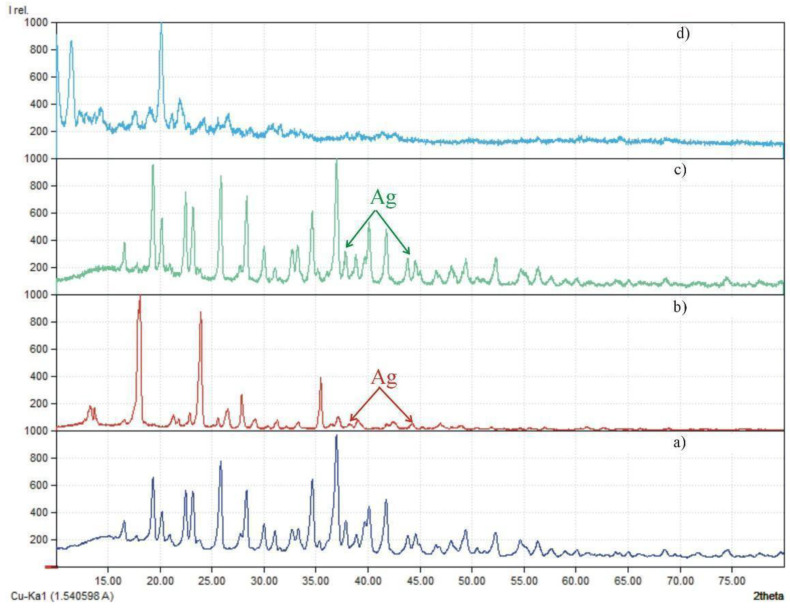
X-ray diffraction patterns of galactose (**a**), Ag NPs (**b**), PID-loaded Ag NPs (**c**), and PID (**d**).

Using the X-ray powder diffraction pattern of galactose, a Rietveld refinement analysis was performed. The proposed cell parameters in the monoclinic space group P 2/m were a = 9.75166 Å, b = 15.63694 Å, c = 7.88817 Å, β = 104.532^o^ (cell volume: 1164.35 Å^3^). The XRD analysis supports galactose’s role in the synthesis of Ag NPs (Figure 6). XRD patterns of pure galactose (Figure 6a) and PID (Figure 6d) revealed multiple distinctive peaks in the 2θ area as a result of their crystalline structure. The X-ray diffraction pattern of PID-loaded Ag NPs showed the reflections for silver at 2θ = 37.80° and 43.84°. We were able to refine the X-ray diffraction pattern of PID, as well. The unit cell parameters found were a = 18.24746 Å, b = 5.24937 Å, c = 9.32495 Å, and β = 124.144°. The space group is P 2/m. 

Compared to the typical galactose XRD pattern, the Ag NPs pattern was altered (Figure 6b). The reflections for silver were measured at 2θ = 37.80° and 43.84°. The 1 1 1 plane, which is associated with the spherical structure of silver [89], corresponds to the strong Bragg reflection at 38.16°.

### 3.2. Phenindione Adsorption onto Galactose-Loaded Nanostructure by DFT

**Figure 7:**
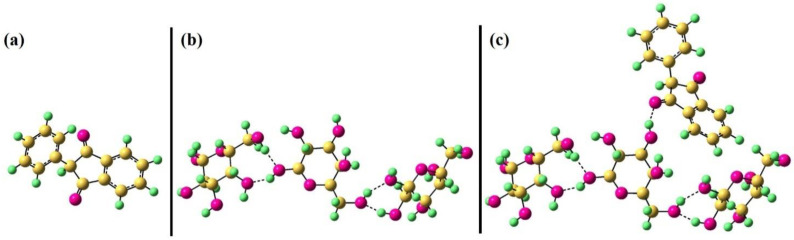
(**a**) The optimized structure of PID, (**b**) nanostructure, and (**c**) PID on nanostructure.

At first, the PID molecule and nanostructure were optimized separately, then the PID drug was placed on the nanostructure, and optimization was performed. The length of the nanostructure was chosen to be 19.11 Å, and its structure consists of 20 carbons, 16 oxygen, and 36 hydrogens. The optimized geometry of PID, nanostructure, and PID-nanostructure is demonstrated in Figure 7. The PID molecule was first situated in various locations on the external surface of the nanostructure with diverse orientations. The ideal and most stable geometry of the PID on the nanostructure is shown in Figure 7c. As can be seen from this figure, the galactose molecules are held together through hydrogen bonds. In addition, the PID molecule interacts with one of the galactose molecules through a weak hydrogen bond. The estimated value of the adsorption energy (E_ads_) for PID on nanostructure is obtained as −0.438 eV (Table 1). The PID’s adsorption energy is negative indicating that the adsorption of this drug on nanostructure is exothermic. The interaction of the PID molecule with the nanostructure is weak due to the fact the E_ads_ value of PID on the nanostructure is small and the presence of a weak hydrogen bond between this molecule and the surface of the nanoparticle. As a result, galactose nanostructure can be utilized as a drug delivery system for the phenindione molecule.

**Figure 8:**
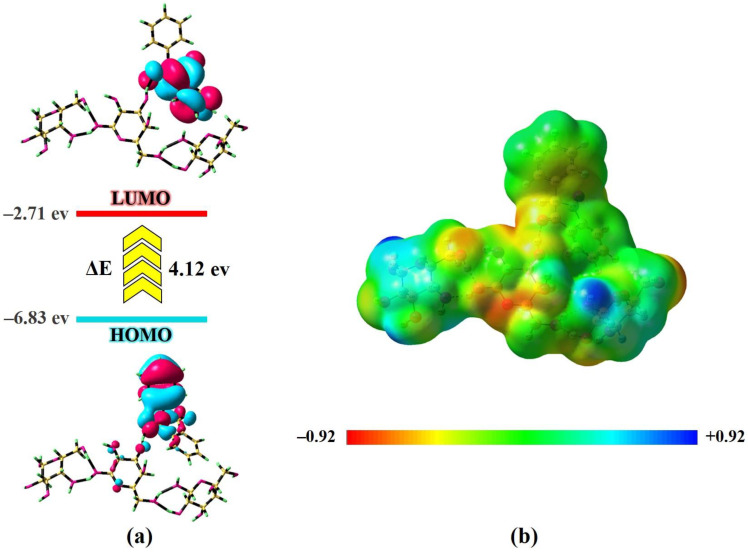
(**a**) HOMO–LUMO diagram of PID on nanostructure, and (**b**) MEP surface of PID on nanostructure.

The quantum descriptors including HOMO-LUMO energy gap (ΔE = E_LU–MO_ − E_HOMO_), absolute hardness (η = (E_LU–MO_ − E_HOMO_)/2), absolute softness (σ = 1/η), chemical potential (P_i_ = −χ), global electrophilicity (ω = P_i_^2^/2η) and absolute electronegativity (χ = −(E_HOMO_ + E_LUMO_)/2) [90,91] were calculated and are summarized in Table 1. The stability and chemical reactivity of a surface or molecule can be investigated using the HOMO/LUMO energy gap. The low-energy gap indicates high chemical reactive nature and vice versa [92,93]. According to Table 1, ΔE value for PID on nanostructure is lower than free PID or nanostructure suggesting this complex possesses higher chemical reactivity than others. In addition, a high ω value illustrates that a compound is more capable of interacting with biological macromolecules such as DNA [94]. A high ω value for PID on nanostructure means that this complex has a high biological activity. Figure 8a demonstrates the HOMO-LUMO energy level for PID on the nanostructure. As clear, the electron density of HOMO is situated in the phenyl group of the PID molecule, and the electron cloud of LUMO is located in the indandione group of the PID molecule.

MEP (molecular electrostatic potential) surface is estimated for PID on nanostructure using the DFT approach and represented in Figure 8b. This surface is beneficial for illustrating the distribution of charge within the system. The MEP utilizes various colors in a 3D scheme including red and blue color. The blue color denotes the electron-deficient zone (positive charge), and the red one demonstrates the electron-rich area (negative charge) [95]. Based on Figure 8b, it can be predicted that the interaction between PID and nanostructure leads to charge transfer from PID molecule to the nanostructure. The charge transfers between phenindione and nanostructure result in a weak interaction. Therefore, the result of MEP also confirms that the galactose nanostructure can act as an appropriate drug delivery system for PID.

### 3.3. Parametric Drug Release Optimization

To find out how the ratio of Ag NPs to the drug molecule would affect the drug release and biological activity, different amounts of PID were used in the preparation of the solutions. The most popular method of medication is oral. Oral medication, compared to intravenous treatments, is easier to take, less painful, and less expensive. A drug’s oral bioavailability will be hampered if its water solubility is too low. Nanocarriers can be used to resolve such solubility issues [96]. The influence of Ag NPs to drug ratio was evaluated utilizing an in vitro approach with dialysis bags in order to get insights into the drug release trends of PID-loaded Ag NPs [97,98]. Due to a lack of universal, accepted practices, this is a typical technique to learn about innovative drug delivery systems [99]. To determine the in vivo–in vitro correlation of nanoparticle formulations, as well as to direct the development and quality control, drug release profiles from dialysis-based assays are used [100,101]. 

Profiles of PID-molecules only (controls) were compared to those seen in the presence of Ag NPs to determine the drug release properties of the PID-loaded Ag NPs. Different Ag NPs to drug ratios (1:1, 1:5, 1:10, 1:20, and 1:50) were utilized to examine how the ratio might impact the drug’s release. One drug control per ratio was made using an ethanol solution of PID. 

Under normal physiological conditions, the pH value of blood is 7.4. To simulate the physiological conditions, the drug release study was carried out in a neutral medium (PBS, pH 7.4). The solution of drug-containing Ag NPs was kept in a cellulose dialysis bag with a molecular weight cut-off of 12,400 Da. The pores in this cut-off were big enough to trap the Ag NPs inside the membrane while yet allowing the medicine to escape from the dialysate. Since the calibration curve for PID was linear, it was possible to conduct the quantification tests. Therefore, determining the drug release in the first 6 h was crucial for our investigation.

**Figure 9:**
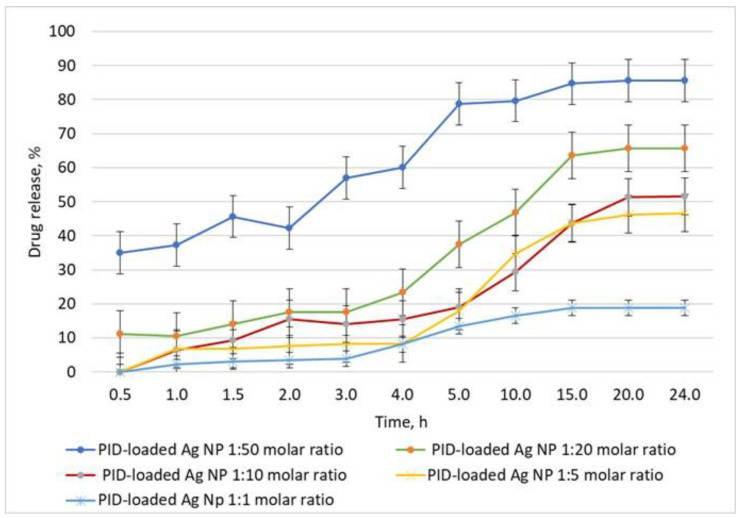
Drug release concentrations of PID-loaded Ag NPs in molar ratios of 1:1, 1:5, 1:10, 1:20, and 1:50.

During the 24 h period, drug release was measured. We found that the absorbance intensity rose with time during the initial 5 to 10 h of the drug release experiment, indicating an increasing concentration of the PID in the dialysate (Figure 9). 

The obtained data showed that the drug release pattern changed a little after the fifth hour, but the rate of increase in absorbance intensity remained constant until the 24th hour. Therefore, in the 24th hour, the maximal concentration of PID had been released in the dialysate, proving that the drug release was complete under the given conditions and that the concentration of the released drug was stable. Due to the existence of a galactose coating and the effectiveness of the drug loading for Ag NPs, the concentration for PID-only samples was greater.

However, at the sixth hour of the drug-release experiment, PID-loaded Ag NPs with a molar ratio of 1:50 demonstrated a very good drug release between 80 and 86%, whereas a molar ratio of 1:20 showed 60–68%, while a molar ratio of 1:1 showed roughly 19% drug release. The obtained results suggested the effectiveness of drug loading, as well as the possible application of Ag NPs as a drug delivery system for PID. 

### 3.4. Antimicrobial Activity

One of the most pervasive problems in the world today is antibiotic resistance, and many potent medications have failed to control illnesses. According to the literature, Ag NPs possess antimicrobial activity [48,51,69,102,103]. Thus, we measured the antimicrobial activity of the PID-loaded Ag NPs compared to PID only. The antimicrobial activity was tested in vitro against human pathogenic bacteria and economically relevant phytopathogenic fungi. In our experiments, six Gram-positive bacteria, six Gram-negative bacteria, two yeasts, and six fungi were used. The inhibition zones of bacterial and fungal growth caused by the novel compounds are outlined in Table 2. The methanol used as a solvent for the samples did not show any antimicrobial effect.

We observed that the PID, as well as PID-loaded Ag NPs, exerted very good activity against Gram-positive bacteria including *Micrococcus luteus* 2YC-YT, *Staphylococcus aureus* ATCC 25923, *Bacillus amyloliquefaciens* 4BCL-YT, Gram-negative bacteria including *Salmonella enteritidis* ATCC 13076, and modest activity against the other bacteria and fungi.

The obtained data showed that in general antimicrobial activity of PID is higher than drug-loaded Ag NPs. We can explain this fact with the time needed to release the drug from the Ag NPs. These observations were also confirmed by anticoagulant and spasmolytic activity measurements.

### 3.5. Cytotoxic Activity

A series of MTT experiments were conducted against normal murine fibroblast cells, malignant human cell lines of leukemic (LAMA-84, K-562), and urothelial bladder carcinoma (T-24) origin to accommodate the cytotoxicity of the target compounds. The results of the antiproliferative assays are presented in Table 3. According to the obtained data, cell growth of both normal and malignant in vitro cultures tends to be unaffected by free PID and the unloaded galactose-assisted Ag NPs (IC50 values invariably exceeding 200 µM), indicating favorable biocompatibility. However, a moderate enhancement of PID cytotoxicity was established in the PID-loaded Ag NPs formulation (particularly in the suspension-growing leukemic cell lines LAMA-84 and K-562), possibly due to improved cellular kinetics and intracellular delivery of the phenindione derivative. Moreover, a number of studies reported on the inhibitory effect of indandione analogues on various kinases and other enzymes (e.g., akt, braf, PI3K, SIK2, among others) [104,105,106], which may account for the antiproliferative activity of these compounds in the tested leukemia models, bearing in mind their BCR-ABL positive phenotype. Nevertheless, the growth-inhibitory potential of the drug-loaded Ag NP is twice as low in the normal fibroblast cellular system, displaying a certain selectivity towards the leukemic cells with elevated constitutive kinase activity.

### 3.6. Anticoagulant Activity

All over the world, venous thromboembolism is the major cause of cardiovascular mortality, next in rank to myocardial infarction and stroke [107,108]. This clinical situation affects patients in various settings and age groups, including children [109,110]. The most prevalent manifestation of venous thromboembolism is deep vein thrombosis, the expression of which may manifest in its most severe form known as acute pulmonary thromboembolism [111,112]. The major treatment for both situations involves the administration of full anticoagulation, thus minimizing the recurrence of venous thromboembolism. One homeostatic mechanism that stops continued bleeding after tissue injury is coagulation. It happens when platelets, plasma clotting factors, and injured tissue interact. The fibrinolytic cascade, which controls fibrin and fibrinogen breakdown and minimizes excessive thrombus formation, counterbalances the coagulation cascade. The coagulation cascade, the fibrinolytic cascade, and the platelet function interact dynamically to produce normal clotting and hemostasis [113].

The capacity of anticoagulants to prevent the production of vitamin K-dependent clotting factors (II, VII, IX, and X) causes their anticoagulant effect. Warfarin, for example, shows its anticoagulant effect 8–12 h after oral or intravenous administration. [13]. Anticoagulants stop the blood coagulation process. Anticoagulant therapy is primarily focused on preventing the development of blood vessel clots, which are the main cause of death in thromboembolic disorders. In our experiments, for screening coagulation tests, a platelet-poor plasma was used. We observed that PID itself showed no coagulation. The measured PT, APTT, and fibrinogen, as well as the reference values, are shown in Table 4.

When PID was tested for its anticoagulant activity, it showed no coagulation. That means that the compound can prolong the coagulation of blood for a longer time causing bleeding in patients. Bleeding is a life-threatening complication.

Drug-loaded Ag NPs, on the other hand, showed that PT (sec) and APTT (sec) are approximately 1.5 times longer than the normal values. Only the molar ratio of 1:50 exerted no coagulation, as PID did itself for any of the molar ratios used. This could make the Ag NPs better anticoagulants than PID itself.

### 3.7. Ex Vivo Experiments on SM Activity

The intrinsic biological activity of substances could be studied both in vivo and ex vivo using isolated tissues that are still functionally active. The ex vivo approach, which is frequently used to assess the potential biological activity of recently synthesized experimental compounds and approved pharmaceutical drugs, is carried out on isolated tissues responsive to physiological stimuli [114]. Because they may still create active tension when separated from the body, SM cells were a natural choice for our study’s platform for ex vivo contractility evaluation [115,116]. Many internal organs primarily have SM tissue. It is a complex superposition of bioelectrical (slow wave with characteristic frequency and amplitude) and contractile activity (CA) (tone, frequency, and amplitude of spontaneous or induced muscle contractions), which can be measured isometrically in isolated tissues [117,118,119] and related to the motor activity of the stomach.

The primary function of smooth muscle is contraction. On ex vivo conditions smooth muscles can preserve their elastic properties for up to 10 h and show a significant change in their tone, frequency, and amplitude of contraction under exogenously administered pharmacological agents [120,121]. 

**Figure 10:**
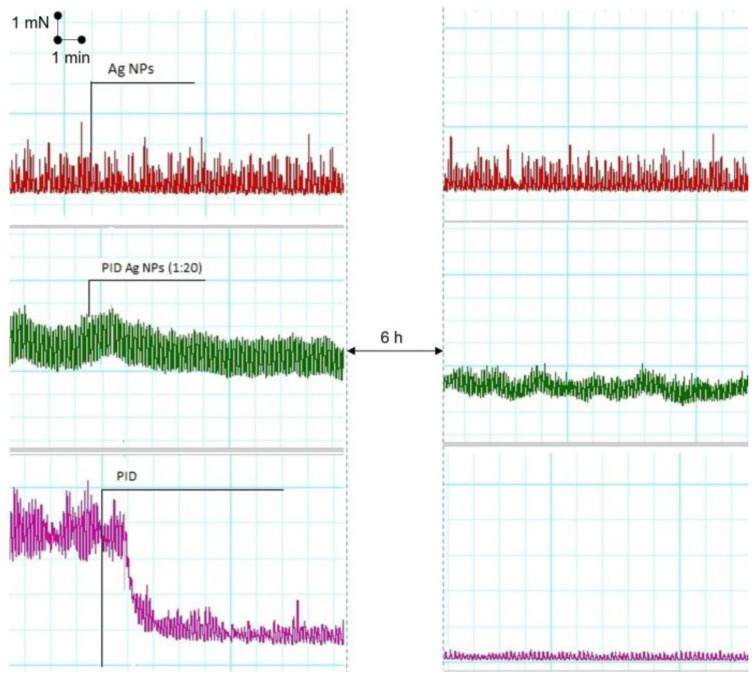
Representative tracings of gastric SM activity parameters before and after application with Ag NPs, PID, and the most pronounced effect of PID-loaded Ag NPs (1:20) at concentration 5 × 10^−5^ mol/L.

The isolated tissue bath experiment is a conventional pharmacological technique for evaluating concentration-response correlations in a range of contractile tissues. Pharmacologists and physiologists continue to utilize this method, even though it has been around for more than a century, due to its versatility, simplicity, and reproducibility [122]. The isolated tissue bath continues to be a significant part of drug development and fundamental research because it enables the tissue to behave like a tissue. Since it permits the tissue to behave like a tissue, the isolated tissue bath continues to be a crucial component of drug development and fundamental research. The ability of the living tissue to function as a whole organ with physiologically realistic contraction or relaxation is the main advantage of this technology. It also has the advantage of bringing the effects of the drugs under research closer to how they would act in the body by allowing for the calculation of important pharmacological variables while retaining tissue function [122]. Previously [123,124,125,126,127], we applied the isolated tissue bath for ex vivo SM activity investigation of different compounds. Thus, we applied the same technique to the exogenous administration of 5 × 10^−5^ mol/L PID-loaded Ag NPs (n = 7), compared to Ag NPs (n = 6) and PID only (n = 6). We found that a single administration of Ag NPs showed no change in smooth muscle spontaneous contractile activity (SCA) for about 6 h (Figure 10). After the 6th hour, we found that the acetylcholine response slightly decreases. 

According to González et al. [128] Ag NPs modify the contractile activity of ACh through nitrogen oxide production and possibly induce smooth muscle hyperreactivity. The obtained data (Table 5) showed that under the same experimental conditions in the tissue bath, PID showed a rapid significant relaxation response with a pronounced change in the tone and amplitude of spontaneous contractions. 

The maximum tonic relaxation appears approximately after 10.47 ± 0.22 min and persists with the same force for 6 h. No change was observed in the ACh response before and after administration of the PID, which indicates that the main neurotransmitter pathway is not affected, unlike Ag NPs. This data confirmed the fact that indane and its analogues showed not only anticoagulant activity but can also affect smooth muscles [129]. When PID-loaded Ag NPs were applied, we found they affected the tone and the amplitude of contractile activity the same way as PID did (Table 5). For comparison, the maximum effect of PID-loaded Ag NPs (1:10; 1:20; 1:50) at concentration 5 × 10^−5^ M was a relaxation with a strength, which was approximately two times lower than the relaxation evoked by 5 × 10^−5^ M PID on the same tissues. After the sixth hour, the effect reaches its peak, which confirms our hypothesis that the nanoparticles release the PID gradually. PID-loaded Ag NPs (1:1 and 1:5) at concentration 5 × 10^−5^ M did not affect the SCA parameters of gastric SMPs. Acetylcholine did not significantly change after the 6th hour, demonstrating that the Ag NPs have already released the maximum amount of PID. Thus, the obtained data correspond to the dialysis drug-release experiments. We assumed that the effects of all PID-loaded Ag NPs on the contractile processes are probably caused by a specific trigger action of the nanoparticles they exert in gastric SM tissue.

## 4. Conclusions

Rapid synthesis and stabilization of silver nanoparticles as drug-delivery systems for PID was applied for the first time. The size range for the produced Ag NPs is determined by using various approaches. Galactose was used as a capping and reducing agent. DFT calculations illustrate that the interaction of PID onto nanostructure is weak, suggesting that galactose-loaded nanostructure can be used as a drug delivery system for phenindione. MEP surface analysis confines that the interaction between phenindione and nanostructure leads to charge transfer from phenindione drug to nanostructure. Additionally, the drug-release capabilities of Ag NPs were established. The drug release showed very good release properties of the Ag NPs between 5 to 10 h of the experiments. The maximum concentration of PID was reached till the 20th h, proving that the drug release was complete under the given conditions and that the concentration of the released drug was stable. Due to the existence of a galactose coating and the effectiveness of the drug loading for Ag NPs, the drug release concentration for PID-only samples was greater. The drug-loaded Ag NPs were characterized in vitro for their antimicrobial and anticoagulant activity and ex vivo for spasmolytic activity compared to PID only. The obtained data showed that Ag NPs exerted very good antimicrobial activity against Gram-positive bacteria (*Micrococcus luteus* 2YC-YT, *Staphylococcus aureus* ATCC 25923, *Bacillus amyloliquefaciens* 4BCL-YT), and Gram-negative bacteria like *Salmonella enteritidis* ATCC 13076, but a bit lower than PID only showed itself due to the time needed to release the drug from the Ag NPs. These observations were also confirmed by anticoagulant and spasmolytic activity measurements. When PID was tested for its anticoagulant activity, it showed no coagulation. That means that the compound can prolong the coagulation of blood for a longer time, causing bleeding in patients, which can be a life-threatening complication. Drug-loaded Ag NPs, on the other hand, showed approximately 1.5 times higher PT and APTT than the normal values. This could make them better anticoagulants than PID itself. The ex vivo spasmolytic experiments showed that PID expressed a rapid and significant relaxation response with a pronounced change in the tone and amplitude of spontaneous contractions. For PID-loaded Ag NPs in the same experiments, the effect reaches its peak after the fifth hour, which confirmed our hypothesis that the nanoparticles release the PID gradually. PID-loaded Ag NPs (1:1 and 1:5) at concentration 5 × 10^−5^ M did not affect the SCA parameters of gastric SMPs. Acetylcholine did not significantly change after the 6th hour, demonstrating that the Ag NPs have already released the maximum amount of PID.

## Figures and Tables

**Table 1:** The adsorption energy (E_ads_) of PID on nanostructure, electronic energy (E_elec_), HOMO-LUMO energy levels, and additional quantum parameters.

Parameters	PID	Ag NPs	PID-Loaded Ag NPs
E_elec_ (eV)	−19,838.000	−56,172.470	−76,010.908
E_ads_ (eV)	-	-	−0.438
E_HOMO_ (eV)	−6.86	−7.16	−6.83
E_LUMO_ (eV)	−2.73	−0.02	−2.71
ΔE_gap_ (eV)	4.13	7.14	4.12
η	2.06	3.57	2.06
σ	0.48	0.28	0.48
χ	4.79	3.59	4.77
Pi	−4.79	−3.59	−4.77
ω	5.57	1.80	5.52

**Table 2:** Antimicrobial activity of the tested PID vs. PID-loaded Ag NPs.

Inhibition Zones, mm		
Tested Microorganism	PID	PID-Loaded Ag NPs
*Bacillus subtilis* ATCC 6633	17	12
*Bacillus amyloliquefaciens* 4BCL-YT	25	13
*Staphylococcus aureus* ATCC 25923	27	27
*Listeria monocytogenes* NBIMCC 8632	22	15
*Enterococcus faecalis* ATCC 29212	23	17
*Micrococcus luteus* 2YC-YT	30	20
*Salmonella enteritidis* ATCC 13076	21	15
*Salmonella typhimurium* NBIMCC 1672	25	13
*Klebsiella pneumoniae* ATCC 13883	11	-
*Escherichia coli* ATCC 25922	18	13
*Proteus vulgaris* ATCC 6380	14	10
*Pseudomonas aeruginosa* ATCC 9027	18	12
*Candida albicans* NBIMCC 74	11	-
*Saccharomyces cerevisiae* ATCC 9763	9	-
*Aspergillus niger* ATCC 1015	11	13
*Aspergillus flavus*	9	-
*Penicillium chrysogenum*	13	10
*Rhizopus* sp.	11	8
*Fusarium moniliforme* ATCC 38932	11	14
*Mucor* sp.	11	-

Dose 0.6 mg/disk, dwell = 6 mm.

**Table 3:** In vitro cytotoxicity of the tested compounds [µM ± SD] against cell lines of different origin.

Compound/Cell Line	CCL-1	LAMA-84	K-562	T-24
PID-loaded Ag NPs	65.4 ± 8.3	25.3 ± 1.2	27 ± 3.2	53.2 ± 4.7
Ag NPs	>200	>300	>300	>300
PID	>300	>200	>200	>300

CCL-1—murine fibroblast cell line; LAMA-84, K-562—BCR-ABL+ chronic myeloid leukemia; T-24—urothelial bladder carcinoma.

**Table 4:** Anticoagulant properties of PID-loaded Ag NPs.

PID-Loaded Ag NPs (Molar Ratio)	PT, s	APTT, s	Fibrinogen, g/L
1:1	14.1	35	0.91
1:5	13.9	34.9	1.34
1:10	13.7	35.3	1.31
1:20	14.1	35.7	1.25
1:50	no coagulation	43.1	1.31
reference	8–10.8	28.2–31.4	1.91–2.87

**Table 5:** Changes in the parameters of SCA of gastric SMPs under the influence of Ag NPs, compared to PID-loaded Ag NPs (in a molar ratio of 1:1, 1:5, 1:10, 1:20, 1:50), and PID itself.

Compound/Parameter	Tonic Activity (mN)	Strength of The Contractile Reaction (mN)	Frequency (Number of Contractions/min)
SCA (initial reaction)	1.81 ± 0.19	3.40 ± 0.19	4.97 ± 0.04
Ag NPs	1.69 ± 0.03	3.15 ± 0.16	4.93 ± 0.03
SCA (initial reaction)	1.89 ± 0.12	3.00 ± 0.18	4.80 ± 0.09
PID-loaded Ag NPs (1:1)	1.80 ± 0.07	2.88 ± 0.05	4.90 ± 0.07
SCA (initial reaction)	2.03 ± 0.15	3.64 ± 0.21	5.04 ± 0.05
PID-loaded Ag NPs (1:5)	2.07 ± 0.22	2.14 ± 0.11	5.09 ± 0.17
SCA (initial reaction)	1.99 ± 0.11	3.64 ± 0.21	5.03 ± 0.03
PID-loaded Ag NPs (1:10)	0.77 ± 0.09 *	2.14 ± 0.11 *	4.87 ± 0.11
SCA (initial reaction)	1.94 ± 0.07	2.44 ± 0.13	5.07 ± 0.10
PID-loaded Ag NPs (1:20)	0.91 ± 0.08 *	0.98 ± 0.07 *	4.90 ± 0.09
SCA (initial reaction)	1.67 ± 0.14	3.50 ± 1.21	4.88 ± 0.06
PID-loaded Ag NPs (1:50)	0.80 ± 0.14 *	1.73 ± 0.16 *	4.83 ± 0.08
SCA (initial reaction)	1.96 ± 0.09	2.42 ± 0.22	4.90 ± 0.04
PID	0.44 ± 0.07 *	0.13 ± 0.03 *	4.87 ± 0.03

The comparison is between spontaneous effects on CA in Krebs solution before and after the application of testing substances. All data were expressed as mean values ± standard error of the mean (mean ± SEM); * statistically significant differences (*p* < 0.05).

## Data Availability

Not applicable.

## Supplementary Materials

The following supporting information can be downloaded at: https://www.mdpi.com/article/10.3390/biomedicines11082201/s1, Figure S1: ^1^H-NMR spectrum of phenindione, Figure S2: ^13^C-NMR spectrum of phenindione; Figure S3: FT-IR spectrum of phenindione.
