# Supplementary material for: Drug-Delivery Silver Nanoparticles: A New Perspective for Phenindione as an Anticoagulant
Source: Biomedicines. 2023 Aug 4;11(8):2201. doi: 10.3390/biomedicines11082201 (PMC10452578; doi:10.3390/biomedicines11082201)

Drug-Delivery Silver Nanoparticles: A New Perspective for Phenindione as an Anticoagulant

Stoyanka Nikolova [1,*], Miglena Milusheva [1,2], Vera Gledacheva [3], Mehran Feizi-Dehnayebi [4], Lidia Kaynarova [5], Deyana Georgieva [5], Vassil Delchev [6], Iliyana Stefanova [3], Yulian Tumbarski [7], Rositsa Mihaylova [8], Emiliya Cherneva [9,10], Snezhana Stoencheva [11,12] and Mina Todorova [1]

Supplementary Materials:

Figure S1: ^{1}H-NMR spectrum of phenindione page 2

Figure S2: ^{13}C-NMR spectrum of phenindione page 2

Figure S3: FT-IR spectrum of phenindione page 3

Figure S1. ^{1}H-NMR spectrum of phenindione.

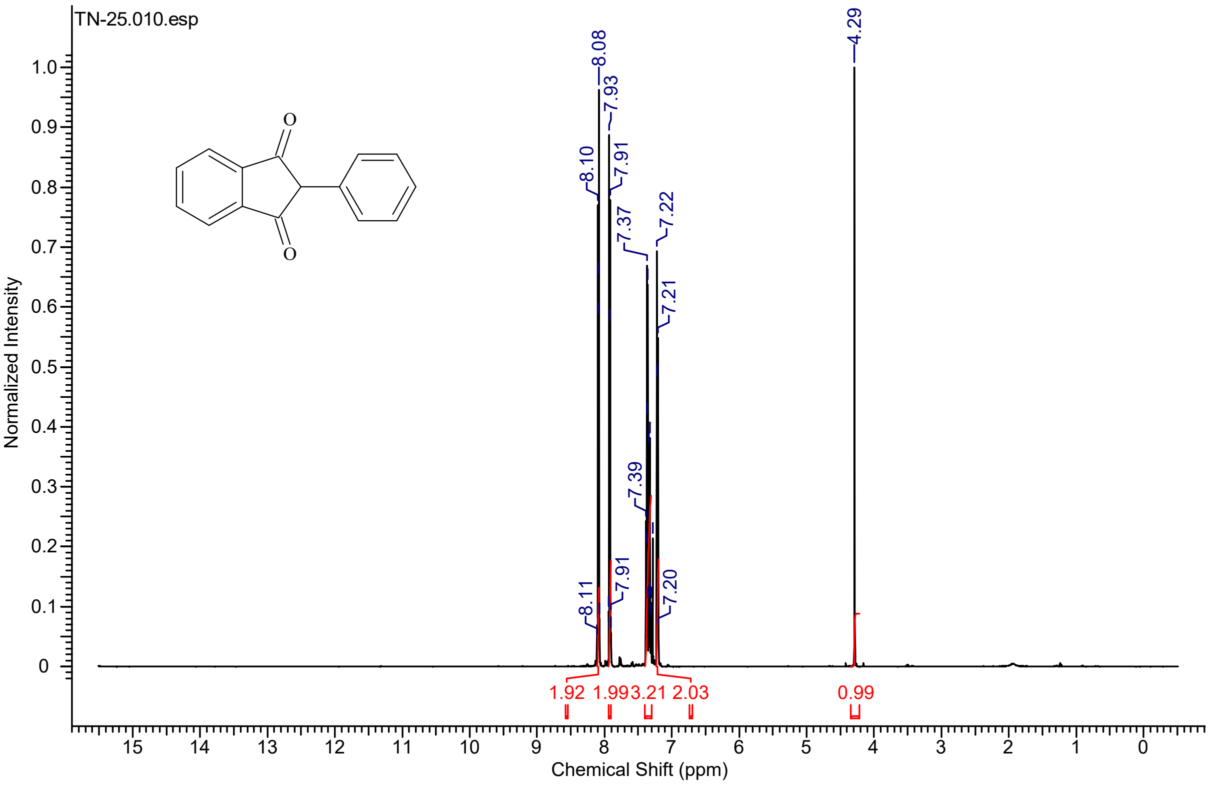

Figure S2. ^{13}C-NMR spectrum of phenindione.

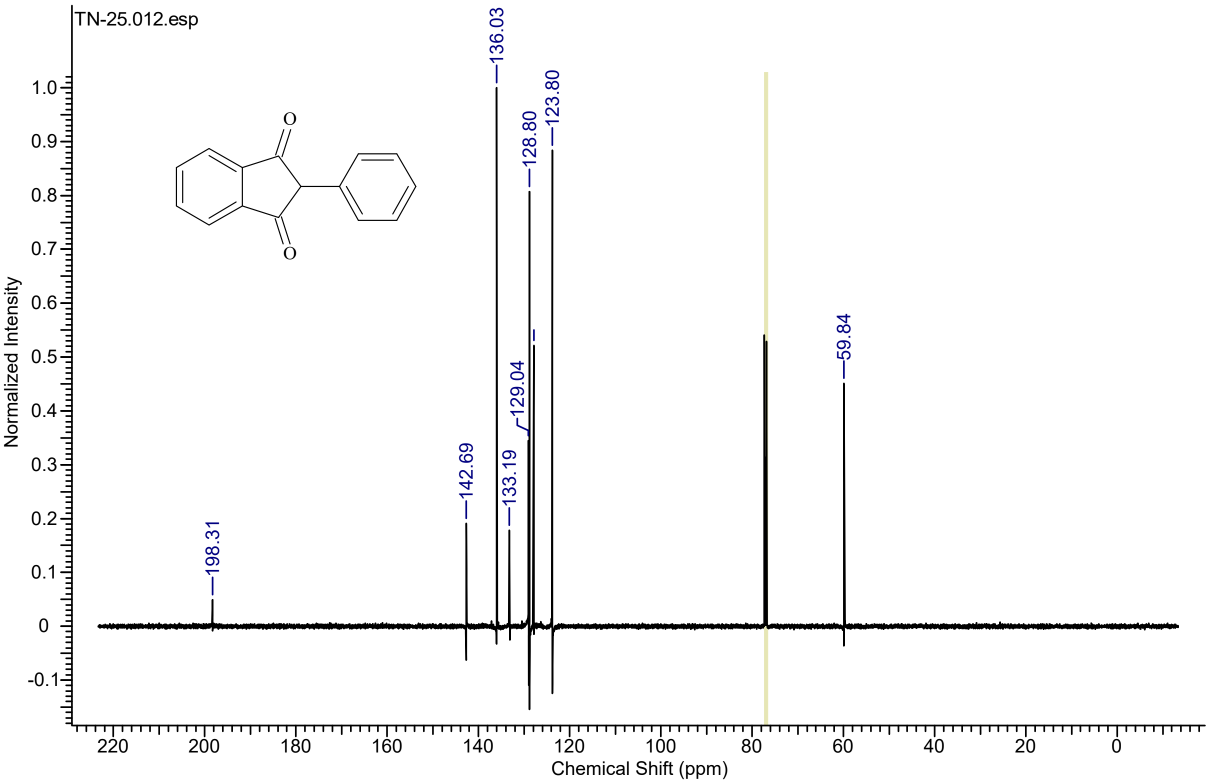

Figure S3. FT-IR spectrum of phenindione.

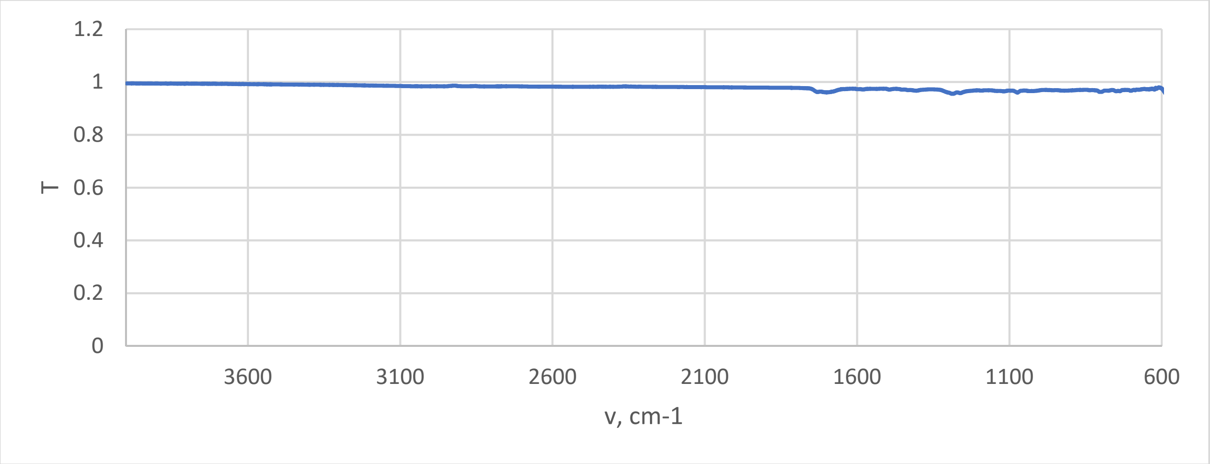

Supplement: Supplementary file 1 [file biomedicines-11-02201-s001.zip › biomedicines-2505888-supplementary.pdf]
